# The changing epidemiology of *Plasmodium vivax*: Insights from conventional and novel surveillance tools

**DOI:** 10.1371/journal.pmed.1003560

**Published:** 2021-04-23

**Authors:** Sarah Auburn, Qin Cheng, Jutta Marfurt, Ric N. Price

**Affiliations:** 1 Global and Tropical Health Division, Menzies School of Health Research and Charles Darwin University, Darwin, Australia; 2 Centre for Tropical Medicine and Global Health, Nuffield Department of Medicine, University of Oxford, Oxford, United Kingdom; 3 Mahidol-Oxford Tropical Medicine Research Unit, Mahidol University, Bangkok, Thailand; 4 Department of Drug Resistance and Diagnostics, Australian Defence Force Malaria and Infectious Disease Institute, Brisbane, Australia; 5 The Australian Defence Force Malaria and Infectious Disease Institute Laboratory, QIMR Berghofer Medical Research Institute, Brisbane, Australia

## Abstract

Sarah Auburn and co-authors discuss the unique biology and epidemiology of P. vivax and current evidence on conventional and new approaches to surveillance.

## Summary points

- Renewed efforts to eliminate malaria have had greater impact on *Plasmodium falciparum* than *Plasmodium vivax*, a reflection of the fundamental differences in the biology of the parasite, its transmission dynamics, and ability to form dormant liver stages.
- The main burden of *P*. *vivax* malaria is in young children residing in remote communities with poor access to healthcare services.
- The decline in *P*. *vivax* malaria has led to an increasing proportion of the parasite reservoir occurring in asymptomatic and low-density *P*. *vivax* infections and heterogeneous patterns of parasite transmission.
- Genetic tools to study the spatial and temporal patterns of *P*. *vivax* transmission in different endemicities and ultrasensitive PCR (uPCR)-based techniques are expanding our knowledge of the magnitude and biology of low-density *P*. *vivax* infections.
- Serology offers alternative ways of detecting recent *P*. *vivax* infections and monitoring of the impact of public health interventions at very low endemicity.

## Introduction

*Plasmodium vivax* remains an important public health burden affecting the poorest and most vulnerable communities of more than 49 endemic countries [1]. Over the past 2 decades, investment in malaria control and elimination programmes has helped to shrink the global malaria map considerably; however, in areas where *P*. *vivax* and *Plasmodium falciparum* are co-endemic, *P*. *vivax* is becoming the predominant cause of malaria [2]. The rising proportion of *P*. *vivax* highlights the greater transmission potential of this species attributable to several biological properties that differ significantly from *P*. *falciparum* (**Table 1**). Key features of *P*. *vivax* confounding its control and elimination include the parasite’s ability to form dormant liver stages (hypnozoites) that can cause relapse weeks to months after an initial infection, the ability to circulate at low peripheral parasite densities while remaining transmissible to the mosquito vector, and the early production of sexual stages (gametocytes) that allow the parasite to transmit prior to clinical presentation and gametocytocidal treatment. *P*. *vivax* has also evolved to survive in a greater variety of *Anopheles* vectors. Collectively, these properties sustain parasite survival and transmission in more extreme climates and confer greater resilience than *P*. *falciparum* against conventional parasite and vector control activities [3].

**Table 1:** Biological properties of *P*. *vivax* that enhance the capacity for transmission and spread.

Biological property	Challenge	Potential solutions
Low-density infections	Clinical illness and transmission occur at low-level parasitaemia, which is hard to diagnose	High-sensitivity molecular diagnostics such as PCR and large volume uPCR
Liver-stage reservoir	Hepatic reservoir is difficult to diagnose and results in recurrent infections that sustain ongoing transmission and confound the assessment of TES	Serological markers to identify individuals with recent exposure and high risk of relapse who can be offered radical cure. Genotyping of infections initially and at the time of recurrence to refine classifications (recrudescence, reinfection, or relapse) in clinical and epidemiological studies
Early gametocytaemia prior to clinical presentation	Enhances transmission prior to antimalarial treatment	Early diagnosis and treatment of clinical illness, prevention of relapses, and transmission interventions such as indoor residual spraying with insecticides and LLINs
Capacity to develop in mosquitoes at low ambient temperatures	Wide range of ecological receptivity, enhancing transmission	Transmission interventions such as indoor residual spraying with insecticides and LLINs

LLIN, long-lasting insecticide treated net; TES, therapeutic efficacy studies; uPCR, ultrasensitive PCR.

**Fig 1:**
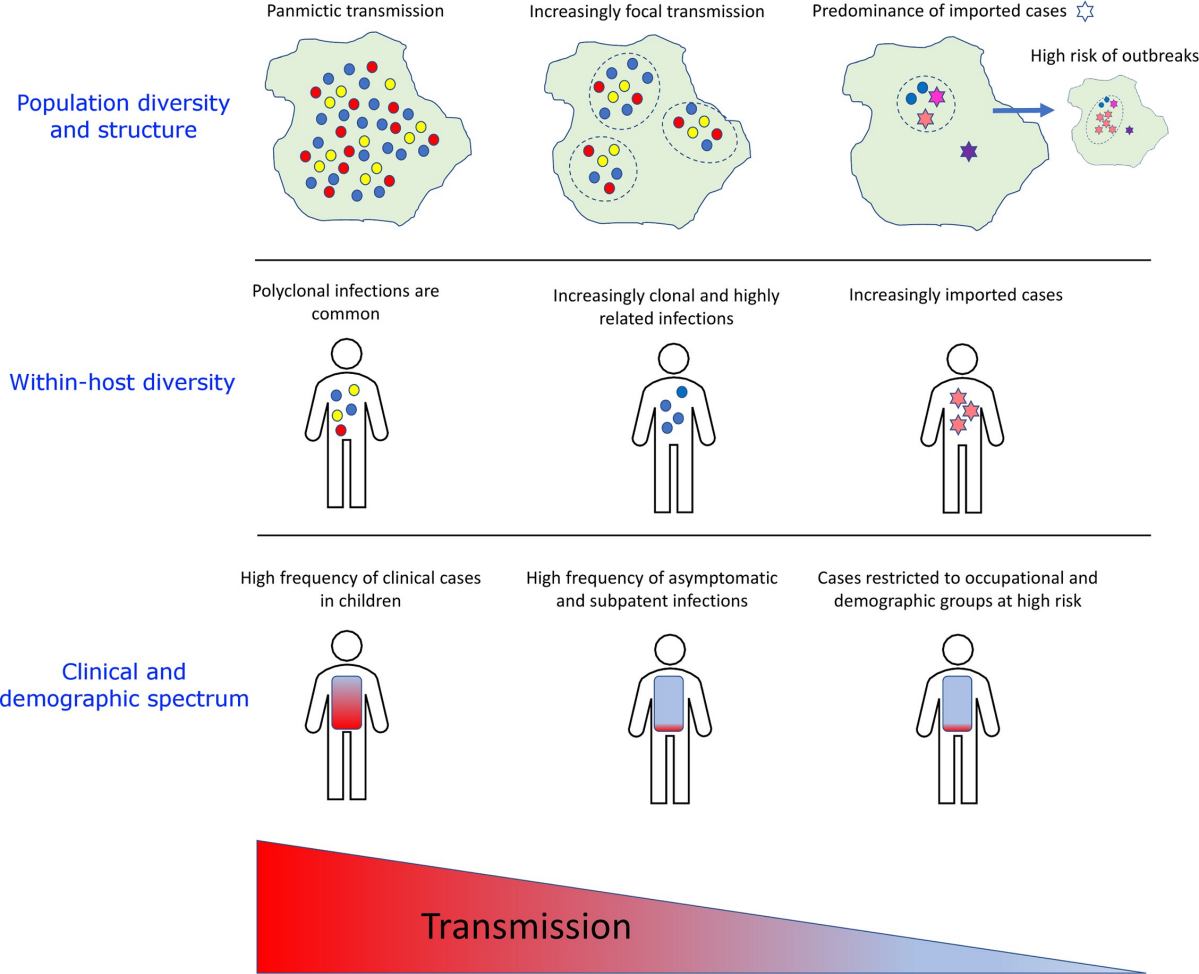
Trends in parasite transmission and diversity, with the spectrum of clinical illness and demographics varying with endemicity. The schematic was inspired by Fig 1 in Wesolowski et al. [36]. As transmission declines, parasite populations become increasingly fragmented into distinct foci and enhanced inbreeding leads to clonal and highly related infections. An increasing proportion of infections are acquired from importations, with high risk of outbreaks. Cases become harder to detect and treat owing to increasingly asymptomatic outcome and SM level. A shift is also observed towards high-risk occupational groups and hard-to-reach populations. SM, submicroscopic.

Despite the relative resilience of *P*. *vivax*, its incidence has declined in most endemic regions, falling from an estimated global burden of 24.5 million clinical cases in 2000 to 14.3 million in 2017 [2]. This decline has been associated with changing patterns in the epidemiology of *P*. *vivax* as endemicity shifts from high and stable to intermediate and pre- and post-elimination levels (**Fig 1**). Examples of the spectrum of national endemicity across 49 *P. vivax*–endemic countries are presented in **Table 2**. However, the mean national incidence measures presented in the table mask marked heterogeneity in endemicity at the micro-epidemiological level. In this review, we discuss the complex dynamics of parasite transmission and spread across this spectrum of endemicity, the epidemiological challenges that they present, and the tools that are being used to monitor them.

**Table 2:** National incidence of *P*. *vivax* malaria in 49 endemic countries.

Incidence of malaria	High	Medium	Low	Very low
API	>10 per 1,000	1–10 per 1,000	0.1–1 per 1,000	<0.1 per 1,000
	Afghanistan Guyana Pakistan Papua New Guinea Solomon Islands South Korea Sudan Vanuatu Venezuela	Belize Djibouti Ecuador Guatemala Honduras Nepal North Korea Panama Somalia Suriname Thailand Yemen	Bolivia Brazil Cambodia Colombia Costa Rica Eritrea Ethiopia French Guiana India Indonesia Laos Madagascar Myanmar Nicaragua Peru Philippines	Bangladesh Bhutan China* El Salvador Iran Kenya Malaysia* Mexico Oman* Saudi Arabia Timor-Leste* Vietnam

Incidences generated from Battle et al. [2], from the estimated number of clinical cases of *P*. *vivax* per annum for the population at risk within each country. These classifications provide only a rough approximation since there is marked heterogeneity in endemicity at subnational level. Quantifying endemicity and its heterogeneity at a local level is critical to guide malaria control activities.

* No indigenous cases since 2018.

API, annual parasite incidence.

## Characterising *P*. *vivax* incidence: The complexity of recurrence

Whereas the incidence of *P*. *falciparum* is driven by recurrent inoculation from infected mosquitoes (reinfections), the incidence of *P*. *vivax* is attributable to reactivation of hypnozoites (relapses). In some areas, antimalarial drug resistance, predominantly to chloroquine, results in schizontocidal treatment failure that contributes to early parasite recurrence (recrudescence) [4]. Radical cure of *P*. *vivax* refers to killing both the blood and liver stages of the parasites to ensure not only clinical recovery from acute infection, but also prevention of subsequent relapses. The frequency and timing of these relapses vary considerably with geographical region, host immunity, and the number of sporozoites inoculated by the mosquito the absolute risk over 12 months ranging from less than 10% in temperate areas to more than 80% in tropical climates [5]. Recurrent parasitaemia in individuals continuing to reside in malaria endemic settings can also be due to reinfection; however, longitudinal cohorts of patients treated with and without primaquine radical cure suggest that these account for less than 30% of all recurrences [6].

## High and stable endemicity

In areas of high *P*. *vivax* transmission and relapse periodicity, nonimmune individuals can have recurrent episodes of malaria every few weeks, a similar force of infection as that seen in areas hyperendemic for *P*. *falciparum*. Recurrent bouts of malaria result in a cumulative risk of anaemia, childhood malnutrition, and a rise in associated morbidity and mortality [7]. However, recurrent *P*. *vivax* infections in early life result in a faster acquisition of immunity than that following *P*. *falciparum*, non-sterilising immunity suppressing clinical disease, and a lower risk of illness in older individuals [8]. In these areas, the primary goal of malaria control programmes is to reduce clinical cases and ongoing transmission of the parasite, largely through vector control measures, widespread access to early diagnosis of patients with clinical illness, and provision of effective antimalarial treatment. Effective surveillance tools to monitor treatment efficacy and progress in transmission reduction are critical.

### Monitoring the impact of public health interventions

Conventionally, the surveillance of *P*. *vivax* malaria has relied upon reporting of the prevalence and incidence of *P*. *vivax* infection, identified by light microscopy (LM) or rapid diagnostic tests (RDTs). In highly endemic settings, case detection is generally passive, focused on diagnosing individuals with clinical illness presenting to health facilities. This approach is constrained by a lack of sensitivity in detecting low-level parasitaemia and failure to detect individuals with asymptomatic infections. Although molecular laboratory methods, targeting parasite DNA, offer greater sensitivity in quantifying the extent of the parasite reservoir, these methods are not feasible or cost-effective in highly endemic settings. An alternative approach to gauge transmission intensity is quantifying parasite genetic diversity from population-representative samples.

**Fig 2:**
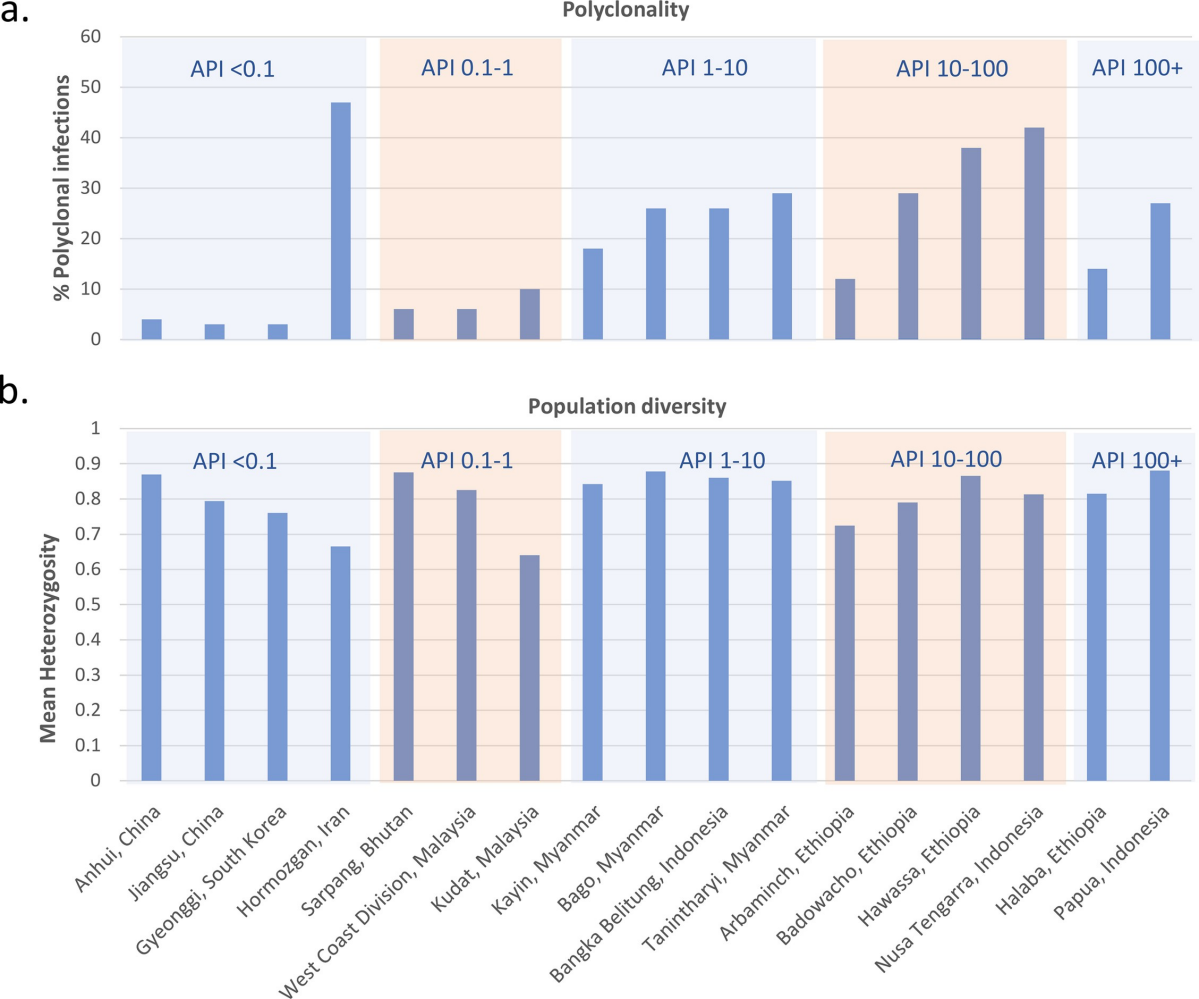
Trends in *P*. *vivax* polyclonality and population diversity across endemic settings. The data were derived from published, open-access online vivaxGEN-MS platform [113]. Sites represent first- or second-level administrative divisions and include areas with a minimum of 15 clinical isolates. Measures were restricted to 3 microsatellite markers: Pvmsp1f3, PvMS16, and Pv3.27 [114]. Polyclonality reflects the percentage of *P*. *vivax* infections with 1 or more markers exhibiting more than 1 allele. The population diversity was approximated using the mean expected heterozygosity. Approximations of the API per 1,000 population were derived from the original publications [16,18,41,91,115–119]. The results demonstrate a general trend of increasing polyclonality with increasing API, with several deviations potentially reflecting factors such as imported cases. In contrast, population-level diversity remains high across endemic settings, potentially reflecting sustained diversity from the liver-stage reservoir. API, annual parasite incidence.

Studies of *P*. *falciparum* populations reveal increasing genetic diversity and prevalence of polyclonal infections with increasing transmission [9]. This trend can be explained by the higher frequency of individuals with multiple infectious mosquito bites in high transmission settings, leading to superinfection (multiple inoculations from different mosquitoes) or coinfection events (a single inoculation with multiple clones) and outcrossing between different clones in the sexual stage of development in the mosquito. However, the correlation between population diversity, polyclonality, and endemicity is less apparent in *P*. *vivax*, with several studies revealing high polyclonality and population diversity even at low endemicity [10,11]. As an example, **Fig 2** illustrates patterns in polyclonality and diversity across a range of endemic settings in a selection of studies that all used the same set of microsatellite markers, revealing several low endemic sites with high diversity. Despite low endemicity, high diversity and polyclonality have been documented in Iran (65% polyclonal) and Sri Lanka (69% polyclonal), potentially reflecting sustained infection diversity from imported cases [12,13]. Genome-wide studies from South American populations have revealed similar trends of extensive genetic diversity in low transmission settings, potentially reflecting a complex composite of historical events and more recent epidemiological patterns [14,15]. Conversely, longitudinal studies reveal a positive correlation between *P*. *vivax* diversity and endemicity. In Sabah, Malaysia, where *P*. *vivax* was eliminated in 2020, there has been a marked decline in both population diversity and polyclonality (50% to 8% from 2010 to 2015) [16]. Whereas in the highly endemic setting of Papua, Indonesia, where the incidence of *P*. *vivax* fell by almost 30% over a 9-year period, polyclonality fell from 67% to 35%, despite no change in parasite diversity [17,18]. A more consistent finding across studies is increasing genetic structure with declining incidence, but this is apparent only after a substantial reduction in transmission [16,19,20]. Collectively, these studies highlight the potential utility of genetic metrices to identify moderate to large changes in transmission reduction that can complement conventional malariometric surveillance. However, a better understanding of how historical and more recent migratory and epidemiological factors may have shaped local population diversity will be needed. In addition, further research is needed to assess how genetic indices can be made readily available and provide timely, actionable, and cost-effective information to malaria control programmes [21].

### Monitoring treatment efficacy

Chloroquine remains the most widely used first-line treatment of *P*. *vivax*, but increasing reports of drug failure highlight the importance of surveillance of its clinical efficacy [4]. Several candidate markers of chloroquine resistance in *P*. *vivax* have been derived from orthologues of *P*. *falciparum* resistance loci, population genomic signatures of selection, and, most recently, a genetic cross; however, none of these markers have been validated against clinical outcomes [16,22–26]. A crucial advance in therapeutic efficacy surveys (TES) of *P*. *falciparum* was the ability to differentiate between reinfections (which tend to be genetically different from the initial infection) and recrudescence (which are genetically the same) using genotyping [27]. The challenge is far greater in *P*. *vivax* since recurrent infections can also arise from dormant hypnozoites (relapses), which may be genetically similar or different from the initial infection [28,29]. Recent studies have demonstrated the utility of genetics to disentangle reinfections from relapses by identifying paired isolates before and after treatment that, while being genetically different, demonstrate familial relationships [30–32]. The rationale is that genetically related infections, such as siblings, are more likely to have derived from the same mosquito inoculation than from different inoculations (reinfections) and are therefore more likely to be relapse events. A mathematical model incorporating genetic and time-to-event data in a TES of primaquine anti-relapse efficacy in Thailand revealed only 2.9% treatment failures compared with an unadjusted clinical estimate of 12% [33]. These genetic tools will greatly enhance the interpretation of TES outcomes for both schizontocidal and hypnozonticidal agents.

## Intermediate endemicity

As transmission intensity declines, the parasite population contracts, transmission becomes increasingly heterogeneous even at small spatial scales, and focalised networks of infection are apparent [34]. In these areas, low-level parasitaemia and asymptomatic infections become increasingly important reservoirs of infection that can sustain ongoing transmission, despite falling numbers of clinical cases. Capturing the burden of these low-level and asymptomatic infections is a major priority for control programmes. A better understanding of the spatial heterogeneity in parasite transmission will inform targeted public health interventions, by identifying residual hotspots of transmission and defining the boundaries and connectivity of these foci.

### Identifying foci of infection and their connectivity

Currently, foci of infection are identified using geospatial surveillance and case investigations [35]. Information on the genetic relatedness between parasites provides a more direct link between infections, enabling greater accuracy in characterising spatial patterns of transmission [36]. Spatial studies of malaria that integrate genetic data with information on patient mobility, such as travel history and mobile phone data, can help characterise transmission networks. Such approaches are relatively advanced for *P*. *falciparum* [37,38], but far more complex for *P*. *vivax* due to confounding from multiple and delayed relapses. Recently, studies using genetic data have shed some light on the connectivity between *P*. *vivax* populations at a subnational level, revealing a spectrum of patterns [11]. In Indonesia and the Pacific region, the connectivity between *P*. *vivax* populations in different islands was surprisingly high despite the geographic barrier to gene flow [39–41]. Comparisons between co-endemic *P*. *vivax* and *P*. *falciparum* populations in these and other studies reveal consistently greater connectivity among the *P*. *vivax* populations, suggesting that the hypnozoite reservoir enhances the spread of *P*. *vivax* infections through human travel [39–43]. In parts of the Peruvian Amazon and along the Pacific Coast of Colombia, connected endemic populations referred to as malaria “corridors” appear to enable *P*. *vivax* infections to spread over long distances, some as far as 1,500 km apart [44,45]. Whereas in other areas of Latin America, *P*. *vivax* populations have much lower connectivity [42,46,47]; restricted human movement and local adaptative pressures leading to the frequent replacement of parasite strains increase the geographic differentiation between these populations. Given the complex and often unpredictable patterns of parasite connectivity, comprehensive genetic surveillance frameworks will be needed to understand how *P*. *vivax* parasites are spreading within and between communities [21]. The methods used to characterise parasite connectivity are also an important consideration for malaria parasites, which have an obligate stage of sexual reproduction in the mosquito midgut. Phylogenetic methods are effective for quantifying genetic relatedness between rapidly mutating organisms that do not recombine but are less suitable for malaria parasites [48]. Furthermore the fixation index (*F*_ST_), a classical approach used to quantify connectedness between populations, does not account for recombination and is therefore constrained in its ability to infer fine-resolution connectedness [49]. Recent studies have employed measures of the probability of interindividual identity by descent (IBD) to characterise the relatedness between malaria parasites, revealing more fine-scale insights into malaria parasite transmission networks [50,51]. However, all these methods are challenged by polyclonal infections, an obstacle that will require new statistical innovations.

### The hidden burden of submicroscopic and asymptomatic infections

The peripheral parasitaemia of *P*. *vivax*, at which an individual develops symptoms, is estimated to be between 40 and 2,000 parasites per μl [52–54]. While the pyrogenic threshold of *P*. *vivax* has been proposed to be significantly lower for *P*. *vivax* compared to *P*. *falciparum*, these estimates do not account for a significant noncirculating biomass. While sequestration of *P*. *falciparum* is well recognised, emerging data suggest a hidden reservoir of *P*. *vivax* [55,56].

Expert microscopic examination of a blood film can detect peripheral parasitaemias as low as 25 parasites μl^−1^, a level at which there are approximately 10^8^ parasites in the peripheral circulation of an adult. However, in routine practice field, microscopy can usually only detect >100 parasites μl^−1^ reliably. Over the past 2 decades, molecular-based diagnostic assays have enabled the threshold of detection to be lowered to 0.02 μl^−1^, unveiling a large hidden burden of submicroscopic (SM) and asymptomatic infections [57]. Since individuals with asymptomatic, low-level, or SM *P*. *vivax* parasitaemia are unlikely to seek medical attention and their infections are likely to be missed by LM or RDTs, they generally remain undetected and untreated.

The scale of the challenge for malaria elimination has been highlighted by the application of molecular approaches to cross-sectional surveys and clinical cohorts which reveal a substantial “hidden” reservoir of infection across the spectrum of transmission settings. Key questions pertinent to malaria elimination strategies include the magnitude of this hidden burden and the degree to which individuals with asymptomatic and SM *P*. *vivax* infections are contributing to ongoing transmission.

In areas of intermediate endemicity, individuals repeatedly exposed to *P*. *vivax* malaria, either by reinfection or relapse, develop a degree of premunition that can suppress clinical symptoms despite rising levels of parasitaemia, hence some asymptomatic *P*. *vivax* carriage can be detected by either LM or PCR. Cross-sectional surveys in the Asia-Pacific region have shown that up to 97% of individuals with *P*. *vivax* parasitaemia detected by LM and 100% of SM infections are asymptomatic [58,59]. In a large cross-sectional study in an area of low and seasonal transmission, ultrasensitive PCR (uPCR, with a level of detection of 0.022 parasites μl^−1^) detected asymptomatic infections in almost a third of individuals of whom 70% had parasite densities below 100 μl^−1^ [57]. Importantly, asymptomatic *P*. *vivax* infections have been shown to include gametocyte carriage and be equally infectious to mosquitoes as symptomatic individuals, suggesting significant potential to contribute to parasite transmission [60,61].

The relative burden of SM *P*. *vivax* infections has been estimated by comparing *P*. *vivax* prevalence detected by LM and PCR. Overall, SM parasitaemia accounted for almost 70% of infection, although there was marked heterogeneity in different transmission settings [59,62]. The relative size of this hidden reservoir increases with increasing sensitivity of parasite detection method used and is greatest in areas with lower transmission intensity [59,63].

### The contribution of SM and asymptomatic *P*. *vivax* infections to transmission

The contribution of SM *P*. *vivax* infections to transmission is determined by several factors including the duration of infection, variation in parasite densities, gametocyte carriage, infectivity to mosquitoes, and propensity to relapse. *P*. *vivax* gametocytes have been shown to transmit more efficiently to *Anopheles* than those of *P*. *falciparum* and to be more transmissible at lower parasite densities [64]. However, transmission dynamics may vary significantly between different vector species and thus these comparisons between parasite species cannot be taken as absolute across the spectrum of malaria endemic environments.

In naturally acquired *P*. *vivax* infections, low-level peripheral parasitaemia fluctuates. Although the duration of infection and dynamics of parasitaemia are likely to determine transmissibility, these are difficult to quantify due to the challenges in conducting longitudinal cohorts with frequent molecular sampling and difficulties in distinguishing persisting infections from relapses and new infections.

In an area of low and seasonal malaria in western Cambodia, 24 asymptomatic individuals with *P*. *vivax* monoinfection were followed for 11 months and assessed monthly using uPCR [65]. All of those assessed tested positive for *P*. *vivax* at 3 or more monthly visits, and almost 20% tested positive every month. Interestingly, of the individuals who had parasite densities <100 μl^−1^ at the start of the cohort, two-thirds never had densities >100 μl^−1^ over the subsequent 11 months. Similar trends were observed in a clinical cohort conducted in Lao PDR [66]. These findings suggest that a significant proportion of individuals with SM *P*. *vivax* control their infection, with parasite densities rarely fluctuating above microscopically detectable levels during recurrent or persisting infection; if confirmed, there may be limited transmission of these SM infections to mosquitoes. Importantly, strategies to target low-level *P*. *vivax* infections by identifying individuals with transient rises in microscopic parasitaemia will not detect most *P*. *vivax* infections. Further studies are required to ascertain the dynamics of parasite densities, clinical outcomes, and transmissibility of these *P*. *vivax* infections over time.

*P*. *vivax* gametocyte carriage can be detected by LM and by reverse transcription quantitative PCR (RT-qPCR) of the *Pvs25* transcript [67]. In acute symptomatic illness, *P*. *falciparum* gametocytaemia tends to occur 7 to 14 days after the appearance of asexual stages, whereas individuals infected with *P*. *vivax* who develop clinical illness often present with both asexual and sexual parasite stages in the peripheral circulation. In both symptomatic and asymptomatic infection, there is a positive correlation between asexual parasitaemia and gametocytaemia, although this correlation varies significantly [68]. Studies in rural Amazonia and Ethiopia where more than half of *P*. *vivax*–infected individuals were asymptomatic revealed that 80% to 90% of asymptomatic and SM-infected individuals had detectable gametocytaemia [60,68]. In contrast, gametocytes were detected in only 20% of asymptomatic and SM *P*. *vivax* infections in Peru [69]. In the Solomon Islands and Papua New Guinea, *Pvs25* transcripts were detected in under 20% of individuals with SM infections compared to 41% to 80% of those with microscopic infection [70,71].

Blood from asymptomatic *P*. *vivax* individuals, mostly detected only by PCR, has been shown to infect 1.2% *Anopheles darlingi*, compared to 22% of blood from symptomatic individuals [72]. Similarly, membrane feeding experiments demonstrate that 3.3% asymptomatic/PCR-detected *P*. *vivax* individuals were infectious compared to 32% of asymptomatic, microscopically positive individuals [68]. Based on these data, the authors estimate that in Ethiopia, SM *P*. *vivax* infections contribute about 14% of the infectious reservoir compared to 56% by the asymptomatic, microscopic detected individuals. However, these studies do not demonstrate the degree to which individuals with low-level infection contribute to transmission over a prolonged period of time. The contribution of low-level *P*. *vivax* infections to transmission is likely to be amplified by the number and frequency of relapses in infected individuals with hypnozoites. While individuals with SM *P*. *vivax* infection may control parasite densities at low levels, a *Plasmodium cynomolgi* model suggests that during relapses, there may be a higher proportion of gametocytes [73]. The evidence highlights that to achieve timely elimination of the parasite, greater efforts should be made to identify all individual harbouring parasites and offering them radical cure of all parasite stages.

It should be noted that even though both asexual and sexual parasites are detected by PCR from peripheral blood, this may not reflect the total *P*. *vivax* biomass. A significant number of trophozoites were seen sequestered in the spleen of one, and both asexual stages and gametocytes were observed in the bone marrow of another *P*. *vivax*–infected individual at similar densities as peripheral blood [74,75]. These sequestered parasites may help to sustain parasitemia in peripheral blood for transmission.

## Low endemicity: Pre- and post-malaria elimination

As countries approach the end stages of malaria elimination, an increasing proportion of infections are imported, either across porous borders or from long-distance importations [76]. There is a shift in the populations at greatest risk of malaria infection, for instance, a relative rise of infections in adult males in occupations that increase their exposure to the local vectors or travel to endemic areas [77–81]. Residual pockets of infection are also observed in hard-to-reach communities such as those in mountainous or densely forested regions, with poor road access [81,82]. New technologies, such as mobile mapping tools, have potential to facilitate tracking human mobility and associated malaria transmission networks in these hard-to-reach populations [83]. At this stage of malaria control, the major goals are to diagnose and treat individuals in the residual pockets of endemicity, identify imported cases, and monitor for evidence of local transmission.

### Surveillance of imported cases

Traditionally, imported malaria has been identified and mapped using information from patients’ travel history. However, persistent blood stage infections and long-latency liver stages constrain the accuracy of this approach in *P*. *vivax*. Genetic studies of *P*. *vivax* have shown that infections from different countries can be differentiated, in some areas with just a handful of single nucleotide polymorphism (SNP) or microsatellite markers [84–88]. A recent genomic study across 20 countries identified 28 SNPs that could distinguish imported from local infections in most areas and provides the first online tool with which investigators can map infections to their country of origin [88]. These genetic tools can complement traditional epidemiologic approaches to inform on imported *P*. *vivax* cases and facilitate surveillance and final certification of malaria elimination status.

While progress has been made in enhancing the surveillance of long-distance *P*. *vivax* importations, the resolution of local from imported cases is still constrained in porous border regions. In regions such as Yunnan Province, China, which shares hundreds of kilometres of border with highly endemic Myanmar, border malaria threatens efforts to prevent community transmission [89]. In large stretches of this border region, parasite gene flow is high, with most cases likely to be imported from Myanmar into China [90]. Similar challenges are faced in several other countries, including southern Bhutan, which shares a border with India, where genetic analyses highlight high homology between local and imported infections [91]. In porous border regions, cross-country collegiate strategies are needed to identify and contain shared reservoirs of *P*. *vivax*. Innovative, robust new tools, and multicountry and regional networks bringing countries together to share knowledge and tools will be critical to strengthen these efforts.

### Detecting local transmission at very low case incidence: The role of seroepidemiology

Seroepidemiology is an alternative approach that can inform parasite surveillance beyond that offered by LM, RDT, and genetic-based methods [92]. Antibodies are biomarkers for exposure to infection, with the advantage that their response profiles last beyond a resolved acute infection when parasites are no longer detectable by LM, RDTs, or nucleic acid amplification–based tests (NAATs) [93]. To date, most studies have focused on *P*. *falciparum*, where seroepidemiological data have been correlated with malaria endemicity and been used to identify transmission hotspots, monitor the impact of control interventions, and evaluate elimination status [94–97]. The advent of the new enzyme-linked immunosorbent assays (ELISAs) technology, which allows assessment of antibody responses to single, or multiple antigens at higher throughput, paralleled by the development and application of more sophisticated analytical methods and models, has expanded our seroepidemiological knowledge and offers opportunities to apply this approach to national and regional surveillance. The rate at which conversion to seropositivity occurs and the longevity of the antibody response to a given antigen vary for different targets and is largely dependent on transmission intensity and booster effects by consecutive reinfections [98]. Age-specific prevalence and seroconversion rates of antibodies against stage-specific antigenic targets such as *Pf*AMA-1, *Pf*MSP-1_19_, and *Pf*MSP-2 have potential to define seasonal and long-term transmission trends [99]. Depending on the stage specificity of antigens, estimates of disease exposure and transmission potential can be further refined: Antibody responses to preerythrocytic antigen targets such as circumsporozoite protein (CSP) and liver-stage antigens (LSAs) have been used to predict recent exposure and monitor temporal trends [100]. Furthermore, antibody responses against gametocyte-expressed targets such as *Pf*mdv1 and *Pf*s16 have been used as indicators of gametocyte carriage and hence, transmission intensity [101]. Antibody responses against the *Anopheles gambiae* salivary gland protein gSG6 or the synthetic peptide gSG6-P1 have been correlated with mosquito exposure [94,102]. While these serological markers have been successfully validated at multiple sites in Africa, Asia, and Southeast Asia, other studies suggest that anti-gSG6 targets of local anopheline vectors are more reliable for estimating transmission intensity [103–106].

The use of protein microarrays and/or fluorescent microsphere-based technologies has advanced the field of seroepidemiology by allowing multiplexing a larger number of antigens at high throughput and thus, enabling screening and validating a multitude of candidate antigens. In a recent study of patients from Thailand, Brazil and the Solomon Islands were screened for more than 300 *P*. *vivax* antigens as potential markers of recent exposure; 8 antigens were identified that detected recent exposure with 80% sensitivity and specificity [107]. Since relapsing infections are key to sustaining *P*. *vivax* transmission, it is crucial to identify individuals at high risk of carrying hypnozoites, so that they can be offered radical cure. Currently, this approach focuses predominantly on patients presenting to clinics with vivax malaria but could be expanded to individuals with asymptomatic parasitaemia detected by active screening, detecting carriers of occult hypnozoites. Further work is underway to confirm these findings in different locations with differing transmission intensity and test the utility of simple RDTs in detecting individuals at risk of relapse.

### Is the last parasite standing the most resistant?

While the first reports of chloroquine resistance in *P*. *falciparum* were in the late 1950s, chloroquine-resistant *P*. *vivax* was not reported until 1989 on the island of New Guinea [108,109]. The delayed emergence in *P*. *vivax* has been attributed in part to the early, presymptomatic gametocytogenesis in this species [110]. Early gametocytogenesis may also enhance the persistence in the population of drug-resistant *P*. *vivax* infections once established locally. In the malaria pre-elimination setting of Sabah, Malaysia, a clinical efficacy study of chloroquine revealed that over 60% of patients failed treatment with chloroquine within 28 days [111]. Interestingly, genomic evaluation of *P*. *vivax* infections collected in the same study demonstrated large clonal outbreaks and a high prevalence of drug resistance variants, including the *multidrug resistance 1* gene (*pvmdr*1) Y976F mutation. The latter has been proposed as a marker of chloroquine resistance, although is at best a minor determinant [16,112]. The combination of unstable transmission and high prevalence of drug-resistant parasites in the late stages of malaria elimination presents a significant risk of resurgence, highlighting the need for diligent surveillance of clinical efficacy. Since clinical trials become logistically challenging as case numbers fall, there is a reliance on molecular surveillance, although for this to be applicable to *P*. *vivax*, better markers of antimalarial resistance will need to be identified.

## Summary

As the map of *P*. *vivax* malaria shrinks, there are marked changes in the epidemiology of the parasite that impact upon malaria control activities. Symptomatic malaria is no longer a disease predominantly of children, and there is an increasing proportion of individuals with asymptomatic and SM infection. Transmission patterns become heterogeneous, and imported cases become important obstacles to complete parasite elimination. Several biological properties of *P*. *vivax* that enhance the transmissibility of this species relative to *P*. *falciparum* present major obstacles to elimination. A key operational challenge is identifying the hypnozoite reservoirs of *P*. *vivax* that define the transmission dynamics of the parasite and providing radical cure of all stages of the parasite to infected individuals. In addition to conventional methods, new surveillance tools offer unique insights into the complex and evolving micro-epidemiology of the parasite, although they are currently largely constrained to research-based activities. Standard and uPCR-based methods can help to quantify the hidden *P*. *vivax* reservoir, beyond patients with symptomatic illness. Genetic analyses can reveal the diversity and connectivity between individual infections and populations that can inform malaria control activities. In areas of very low incidence, novel serological methods offer promise for measuring populations exposed to recent infection, at high risk of relapse, so that individuals and populations can be targeted for test and treat strategies and even mass drug administration. Many of these tools are in their infancy, and their cost-effectiveness and operational utility have yet to be defined (**Table 3**), but their development and application to *P*. *vivax* will be critical in achieving the ultimate and timely elimination of the parasite.

**Table 3:** Challenges with conventional and new surveillance methods.

Objective	Approach	Challenges
**Monitoring the impact of public health interventions**	Passive case detection with RDTs or LM	Lack of sensitivity in detecting low-level parasitaemia, failure to detect individuals with asymptomatic infections, and reliance on treatment-seeking behaviour
	PCR-based parasite detection	Not feasible or cost-effective in most endemic settings
	Parasite genotyping to gauge transmission intensity	Utility and cost effectiveness of genetic indices need to be defined in different endemic settings [21]
	Serology	Requires validation of responses in different endemic settings to predict individuals at increased risk of recurrent *P*. *vivax* parasitaemia
**Detecting submicroscopic and asymptomatic infections**	Community surveys using ultrasenstive diagnostics such PCR-based parasite detection and next generation RDTs	High demand on resources and challenges to produce timely results
**Identifying foci of infection**	Geospatial surveillance and case investigation	The liver-stage reservoir constrains accurate linkage between *P*. *vivax* infections
	Parasite genotyping	Genetic data provide a direct link between infections, but the cost efficacy of this approach at large scale needs to be evaluated [21]
**Monitoring treatment efficacy**	TES	Logistically challenging and difficult to classify the origin of recurrences (recrudescence, reinfection, or relapse)
	Genotyping parasite drug resistance markers	No validated molecular markers of the most widely use blood- or liver-stage antimalarial drugs for *P*. *vivax* [112]
	Parasite genotyping to improve recurrence classification	Limited number of clinical isolates are amenable to whole genome sequencing. Parsimonious genotyping and modelling approaches are being developed to characterise the IBD between infections [33]
**Identifying imported cases**	Travel history	Persistent blood stage infections and long-latency liver stages constrain the accuracy of assumptions based on travel history
	Genotyping	Scope for detecting long-distance imported cases, but currently unable to distinguish local from imported cases in porous border regions [88]

IBD, identity by descent; LM, light microscopy; RDT, rapid diagnostic test; TES, therapeutic efficacy studies.

## Abbreviations

CSP: circumsporozoite protein
ELISA: enzyme-linked immunosorbent assay
IBD: identity by descent
LM: light microscopy
LSA: liver-stage antigen
NAAT: nucleic acid amplification–based test
RDT: rapid diagnostic test
RT-qPCR: reverse transcription quantitative PCR
SM: submicroscopic
SNP: single nucleotide polymorphism
TES: therapeutic efficacy surveys
uPCR: ultrasensitive PCR
